# Supplementary material for: Encapsulation of AgNPs in a Lignin Isocyanate Film: Characterization and Antimicrobial Properties
Source: Materials (Basel). 2023 Jun 8;16(12):4271. doi: 10.3390/ma16124271 (PMC10302273; doi:10.3390/ma16124271)
Supplement: Supplementary file 1 [file materials-16-04271-s001.zip › materials-2202884-supplementary.pdf]

Supplementary Materials

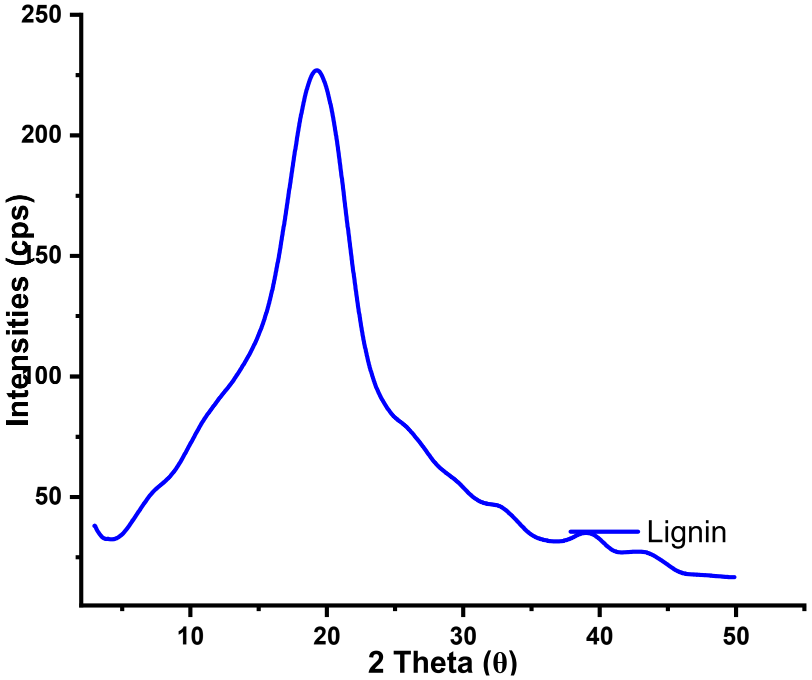

Figure S1. XRD profile of lignin isolated from rice husks black liquor.

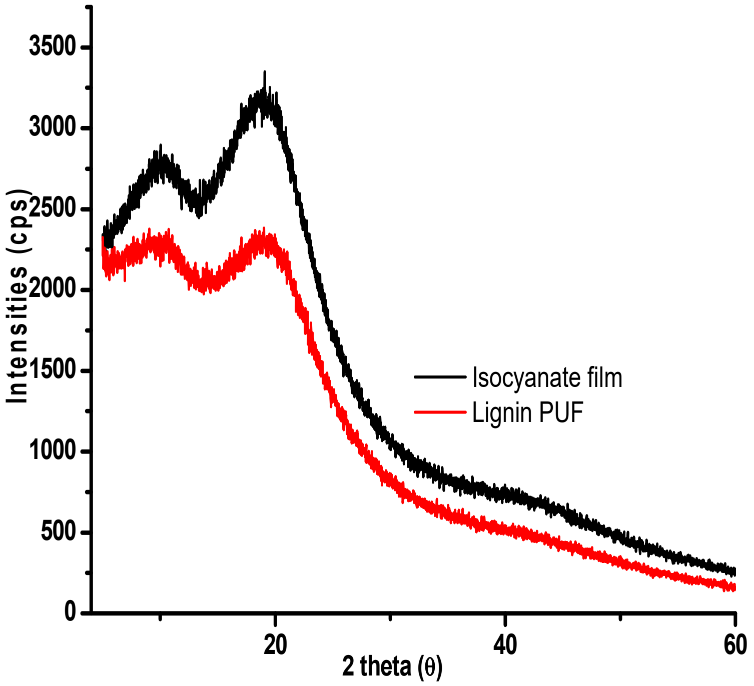

Figure S2. WXRD profile of isocyanate and lignin PUF.
